# No handedness effect on spatial orientation or ocular counter‐roll during lateral head tilts

**DOI:** 10.14814/phy2.14160

**Published:** 2019-07-06

**Authors:** Ariel Winnick, Shirin Sadeghpour, Michael Sova, Jorge Otero‐Millan, Amir Kheradmand

**Affiliations:** ^1^ Department of Neurology The Johns Hopkins University School of Medicine Baltimore Maryland; ^2^ Department of Otolaryngology‐Head and Neck Surgery The Johns Hopkins University School of Medicine Baltimore Maryland

**Keywords:** Handedness, hemispheric laterality, ocular counter‐roll, ocular torsion, spatial orientation, subjective visual vertical, SVV

## Abstract

Although vestibular inputs are bilaterally represented within the cerebral hemispheres, the higher level vestibular functions exhibit hemispheric asymmetries. Previous studies have suggested that such asymmetries are associated with handedness. Here, we studied the impact of handedness (i.e., hemispheric lateralization) on spatial orientation using a subjective visual vertical (SVV) task. We tested 22 right‐handed and 22 left‐handed subjects in upright position, during prolonged lateral head tilts of 20° (~15 min), and after the head returned to upright position. The corresponding changes in torsional eye position were measured simultaneously using video‐oculography. During lateral head tilts, both right‐ and left‐handers had initial SVV biases in the opposite direction of the head tilt (right‐handers: left tilt 3.0 ± 1.3°, right tilt −4.7 ± 1.5°; left‐handers: left tilt 3.4 ± 1.1°, right tilt −4.1 ± 1.0°). The SVV subsequently drifted in the direction of the head tilt, and there was an aftereffect in the same direction when the head was brought back upright. The ocular torsion initially changed in the opposite direction of the head tilt (right‐handers: left tilt 3.8 ± 0.4°, right tilt −3.8 ± 0.4°; left‐handers: left tilt 4.2 ± 0.5°, right tilt −4.5 ± 0.5°), and there were also drift and aftereffect in the same direction as the head tilt. The changes in upright perception and ocular torsion did not differ between right‐ and left‐handers. These findings show no functional laterality, neither in the higher level neural mechanisms that maintain spatial orientation, nor in the lower level mechanisms that generate the ocular torsion response during lateral head tilt.

## Introduction

Vestibular inputs project bilaterally to the cerebral hemispheres, but considerable asymmetry and hemispheric dominance of vestibular‐mediated functions have been reported  in a number of investigations. Functional imaging studies have found dominance of vestibular activity in the right hemisphere of right‐handers and the left hemisphere of left‐handers (Suzuki et al. 2001; Dieterich et al. 2003). Similarly, there is an asymmetry in the “high‐level” modulating effects of cerebral hemispheres on the “low‐level” vestibulo‐ocular functions within the brainstem (Arshad et al. 2013; 2015a, 2015b, 2015c; Nigmatullina et al. 2016). The asymmetric hemispheric contributions also affect perception of spatial orientation, as manifested by the deficits in hemispatial neglect, which are predominantly reported with the right hemispheric lesions (De Renzi et al. 1971; Kerkhoff and Zoelch 1998; Kerkhoff 1999; Gentaz et al. 2002; Saj et al. 2005; Karnath and Dieterich 2006; Funk et al. 2011; Utz et al. 2011; Karnath and Rorden 2012; Braem et al. 2014). These findings suggest that the processing of spatial information can be associated with the handedness‐related variation in brain networks. Such an association raises the question of whether the asymmetric hemispheric contributions are functionally evident with respect to how spatial orientation is modulated by the head or body position.

A key aspect of our spatial orientation is maintaining a stable perception of the world in upright orientation despite continuous changes in the body position within the environment. The neural and behavioral contributions to perception of spatial orientation can be studied by removing spatial cues in experimental settings. When the visual cues are removed, the brain must rely on signals that encode the position of the head and body relative to gravity and the position of the eye relative to the head in order to determine the orientation of external stimuli. This is the basis for a psychophysical task known as the subjective visual vertical (SVV), in which a visual line is used to measure perceived earth‐vertical orientation (i.e., upright perception) (Kheradmand and Winnick 2017). When the head is tilted laterally (i.e., the roll plane), there is a compensatory torsional eye movement in the opposite direction of the head tilt. This ocular counter‐roll (OCR) – in contrast to the vestibulo‐ocular reflexes in the horizontal and vertical planes – is far less than the actual amount of head tilt (gain of about 0.1–0.25) (Leigh and Zee 2015). Thus, during lateral head tilts, the vertical meridians of the eyes deviate from the axis of gravity, and consequently the orientation of the images changes on the retina. This physiological constraint in spatial orientation is reflected by the systematic errors in SVV responses when the head is tilted (Pavlou et al. 2003; De Vrijer et al., 2009; Otero‐Millan and Kheradmand 2016; Kheradmand and Winnick 2017). During large head tilts (e.g., greater than 60°), SVV errors are biased toward the direction of the tilt position. This bias represents the underestimation of upright orientation with respect to the head position and is known as the Aubert or A‐effect (Aubert 1861). At smaller angles (e.g., less than 60°), however, SVV errors are usually biased in the opposite direction of the head tilt position, which represents the overestimation of upright orientation with respect to the head position, known as the Müller or E‐effect (Müller 1916).

The errors of upright perception reflect the function of multisensory neural processes involved in maintaining spatial orientation with changes in the head or body position. In this study, we asked whether there is functional laterality in these processes during lateral head tilts. When a lateral head tilt is maintained, SVV errors change gradually over time, showing that spatial perception can adapt to head tilt position. The common pattern is an SVV drift in the direction of the head tilt, followed by a post‐tilt bias or “aftereffect” when the head returns to upright position, a bias which is in the same direction as the SVV drift (Lechner‐Steinleitner 1978; Wade 1968; 1970; Tarnutzer et al., 2013; Otero‐Millan and Kheradmand 2016). Neither the drift nor the aftereffect correlates with the changes in ocular torsion during or after lateral head tilts (Otero‐Millan and Kheradmand 2016). Such dissociation shows that the drift in upright perception is generated primarily by the neural processes that integrate signals encoding head and eye positions, rather than the actual changes in ocular position within the orbit. Considering these findings, here we measured SVV error, SVV drift, and SVV aftereffect in association with handedness. The corresponding changes in ocular torsion were also recorded along with the SVV responses. In this context, a difference in the SVV measures between right‐handers and left‐handers would indicate a functional laterality in the higher level neural processes that contribute to perception of spatial orientation. On the other hand, a difference in ocular torsion would indicate a functional laterality in the lower level vestibulo‐ocular reflex that drives the OCR during head tilt.

## Materials and Methods

The experiments were approved by the Johns Hopkins Institutional Review Board and informed written consent was obtained from all participants. Forty‐four participants, 22 right‐handed (mean age 28.4 years, 14 female) and 22 left‐handed (mean age 30.4 years, 13 female) were enrolled in the study. All participants were in good health without vestibular, neurologic, or psychiatric illness. Two handedness scales, the Edinburgh Handedness Inventory and the Tapley‐Bryden proficiency test, were completed by subjects to verify their handedness (Oldfield 1971; Salmaso and Longoni 1985; Tapley and Bryden 1985). In the Edinburgh Handedness Inventory, subjects were asked to mark which hand they preferred to use daily in a 10‐item questionnaire. The list of tasks included writing, drawing, throwing, using scissors, brushing their teeth, using a knife, using a spoon, using a broom (upper hand), striking a match, and opening a box (or lid). Accordingly, the handedness was scored based on the responses, using the equation $\frac{R-L}{R+L}$ to calculate a laterality quotient. The Edinburgh handedness laterality quotient for the right‐handers was 80.6 ± 4.6 (mean ± SEM), and −69.6 ± 4.3 for the left‐handers. The Tapley‐Bryden proficiency test utilizes a nonsubjective handedness measurement. In this test, subjects are asked to place dots in small circles as quickly as possible based on their hand preferences in intervals of 20 sec (Tapley and Bryden 1985). Subjects first used their dominant hand, followed by two trials of the nondominant hand, and then finished with one trial of the dominant hand. Accordingly, the number of successfully placed dots (one dot per circle) was measured and scored based on the equation $\frac{R-L}{R+L}$ to calculate a laterality quotient for handedness. The Tapley‐Bryden laterality quotient for the right‐handers was 0.2 ± 0.02 (mean ± SEM), and −0.2 ± 0.02 for the left‐handers.

### Experiment setup

**Figure 1:**
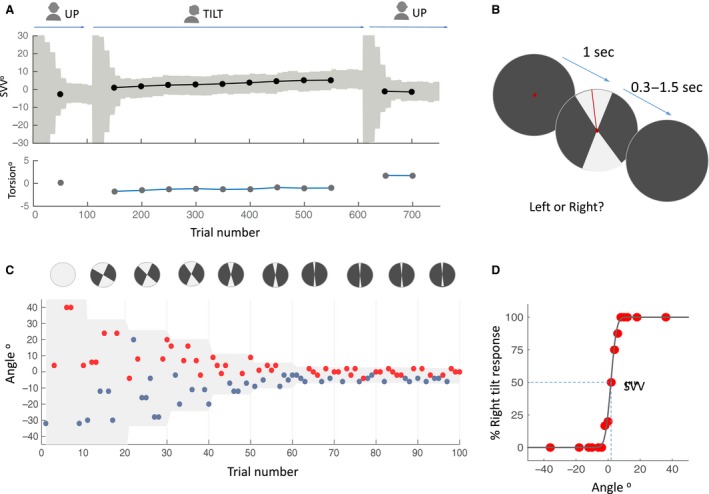
(A) Sample SVV recording during an entire session. Each SVV point is calculated from a psychometric fit to the responses from 100 trials (see D) and each torsion point corresponds with the average ocular torsion during the same block of 100 trials. First, the head is in upright position, then tilted laterally, and finally brought back to the upright position. Every time the head position changed, the paradigm reset and angles were presented starting again at the full range of 360° (light gray shade in C). (B) Subjective visual vertical (SVV) paradigm. In each trial, subjects fixated on a red dot for  one sec before the line appeared. They had 1.5 sec to respond whether the line was tilted to the left or to the right of what they perceived as upright (two‐alternative forced choice or 2AFC). This was done by pressing the left or right button on a controller. The line was presented within a range of possible angles (gray shade) that varied during the experiment (see B). After pressing the button, the line disappeared and the next trial started with a new line orientation. (C) Sample time course of 100 trials with the subject’s responses. Each point represents one trial. The *y*‐axis shows the angle of the line presented and the color indicates the subject’s response for that trial. Left tilt responses are shown in blue and right tilt responses in red. The line angles were presented randomly within a range that started at 360° and then adjusted based on previous responses (illustrated by the light gray sectors in the top circles). At the end of every 10 trials, the center of this range (light gray shade) was set as the SVV value calculated from previous 30 trials. The size of the range was also adjusted every 10 trials by dividing it in half until it reached 8° (±4° around the calculated center), after which it was kept constant for the rest of the trials. Note that for clarity, the figure is simplified with the vertical axis truncated, excluding some of the trials falling outside of the ±45° range. (D) An example of psychometric fit to the responses in the SVV paradigm. The SVV value is determined as the center of the curve (i.e., point of subjective equality), where the chances of right and left responses are equal.

Each subject completed two experiment sessions, one with the head tilted 20° to the right, and one with the head tilted 20° to the left, in a random order across all subjects. We chose 20° lateral tilt as it is large enough to produce SVV errors, yet within the comfortable range of positions to maintain for long recording sessions (Otero‐Millan and Kheradmand 2016). Typically, the head tilt of 20° results in an SVV error in the opposite direction of the head tilt (i.e., E‐effect). The visual line stimulus in the SVV paradigm appeared on a CRT monitor (1280 px by 1024 px) 135 cm away in front of the subject in an otherwise completely dark room. To eliminate all possible visual cues, we set the brightness and contrast levels of the screen to minimum (screen luminance < 0.5 cd/m^2^). The room had no windows and was specially designed to perform experiments in the dark with all walls, floor, and ceiling painted in black color and doors sealed using thick drapes. To further eliminate any potential cues coming from the CRT monitor itself after subjects were dark‐adapted, the monitor was covered by a black cardboard, and the SVV line stimulus appeared in the center of a circular opening. The head was immobilized using a molded bite bar during the experiment sessions. We mounted the bite bar on a rotary motor (Zaber Technologies Inc., Vancouver, BC, Canada) in order to change the head tilt position remotely. The SVV was first recorded in upright position, and after 100 trials the bite bar was tilted remotely to record 500 trials while the head remained in a lateral tilt position (Fig. 1) (see SVV paradigm section for more details). The bite bar was then tilted back upright to record 150 more trials (total of 750 trials). We added a 30‐sec pause in the SVV paradigm after each time the head changed position, to avoid residual effects from canal stimulation during head movement. In all experiment sessions, ocular torsion was recorded simultaneously along with the trials in the SVV paradigm.

### Ocular torsion

We used RealEyes xDVR goggles manufactured by Micromedical Technologies Inc., Chatham, IL, USA and custom software to record eye position. This system uses two cameras (Firefly MV, PointGrey Research Inc., Richmond, BC, Canada) mounted on a pair of goggles to capture infrared images of each eye. Subjects wore these goggles during the entire experiment sessions. To measure torsional eye position, we used a method based on iris recognition and tracking developed by our group (Otero‐Millan et al., 2015). This method can track ocular torsion binocularly in real time at 100 Hz and with a noise level less than 0.1°.

### SVV paradigm

The paradigm was controlled by a custom software written in Matlab (Mathworks) using Psychtoolbox (Kleiner et al. 2007). In order to measure perceived upright orientation, a red line (length: 7.6° of visual angle, width: 0.13°) was presented in random angles around a red fixation dot at the eye level (diameter: 0.33°) in a two‐alternative forced choice (2AFC) task. In each trial, while looking at the fixation dot, subjects clicked a right or left button on a controller to report whether the line was tilted to the right or left of what they perceived as upright (Fig. 1). The fixation dot appeared first, and then after  one sec, the visual line was presented for a minimum of 300 msec and maximum of 1.5 sec until subjects responded. They were given instructions to respond as quickly and accurately as they could and that if the response was not given  within a time window of 1.5 sec, the line stimulus would disappear, in which case they had to click a button to start a new trial. The line orientations were randomly selected within a range that was adjusted in blocks of 10 trials. The line orientations were discretized in steps of 2° including 0°. In each block, five different angle orientations were presented in the top of the visual field (always radiating from the fixation point) and equivalent five angles were presented in the bottom of the visual field. If the subject did not respond within 1.5 sec, the line disappeared, and a new trial started after a button was clicked. In such cases, the missed angle was presented again at a later time within the same block to ensure that all angles were presented equally. At the beginning of the paradigm, the angles were selected randomly from the entire range of 360°, but as the paradigm continued a new range of angles was calculated. This range was centered around the SVV calculated from the responses  of the previous 30 trials (as the 50% point of a logistic regression to the subject responses). The range amplitude also decreased by half until the paradigm reached the ninth block, after which it remained constant at 8° for the rest of the trials. Thereby, the SVV paradigm could adapt and track changes in subjects' perception, and thus it was not biased by making any prior assumption about the SVV value. Every time the head changed position from upright to tilt, the paradigm reset to the starting range of 360° (Fig. 1).

### Data analysis

We measured the following outcomes: (1) SVV in the upright position, (2) SVV at the beginning of the tilt, (3) ocular torsion at the beginning of the tilt, (4) SVV drift, (5) ocular torsion drift, (6) SVV aftereffect, and (7) ocular torsion aftereffect. The reaction times in the SVV paradigm were also measured throughout the experiments. SVV was calculated by fitting a psychometric curve to the trial responses using a cumulative Gaussian function and a generalized linear regression model (Matlab fitglm with probit link function). The SVV value was the angle at which the probability of a left or right response was 50% (point of subjective equality). The SVV precision was calculated as the standard deviation of the cumulative Gaussian fit of the psychometric curve. In order to compare ocular torsion and SVV responses, we first calculated the average torsional position of the two eyes during each trial in the SVV paradigm. Then we calculated the average ocular torsion within a window of 100 trials, and a psychometric curve was fitted to the responses from these 100 trials to calculate the SVV value. This window then advanced in steps of 50 trials to obtain more SVV and ocular torsion values. The first 50 trials were discarded as the range of angles in these initial trials was not narrow enough to get a reliable SVV value. We used a simple linear regression to measure the drift over time and to estimate the rate of change for both SVV and ocular torsion. To calculate correlations across subjects, we first averaged the values for the right and left head tilts and then used Spearman method to obtain the correlation coefficient. For comparisons within the left‐handed and right‐handed groups, we used paired *t*‐tests with a significance level of 0.05. To compare the results between the left‐handed and right‐handed groups, we used repeated measures ANOVA with head tilt as a within‐subject factor and handedness as an across‐subject factor. We tested for an overall bias caused by handedness and the interaction between the handedness and head tilt in our results (i.e.,  whether the handedness changed the biases induced by the head tilt).

## Results

**Figure 2:**
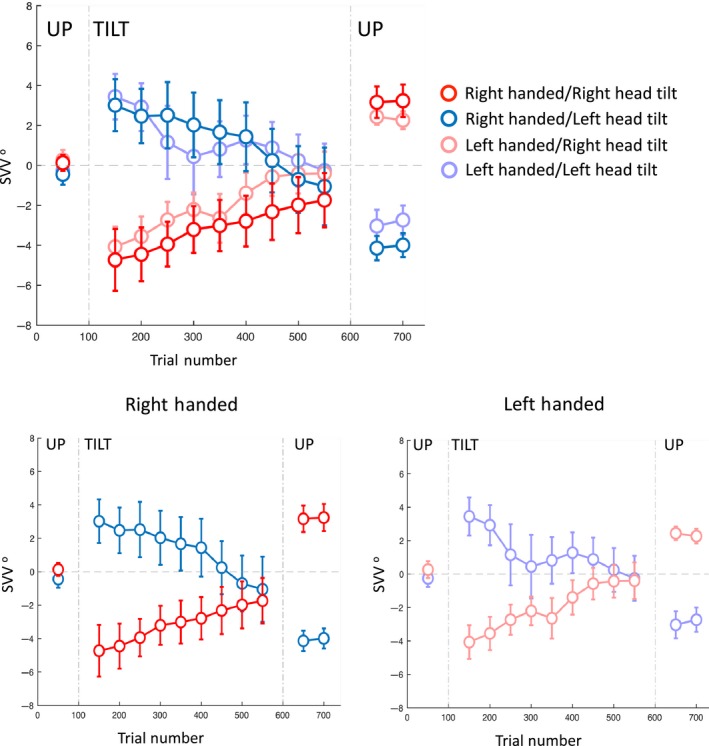
Average SVV during head tilts to the right and left for both right‐ and left‐handed subjects are shown together (top panel) and separately (bottom panels). Each point corresponds with the SVV calculated from responses within 100 trials. The gaps in the data correspond with the first 50 trials in the new head position where SVV estimates were not reliable and were discarded. Error bars indicate SEM.

**Figure 3:**
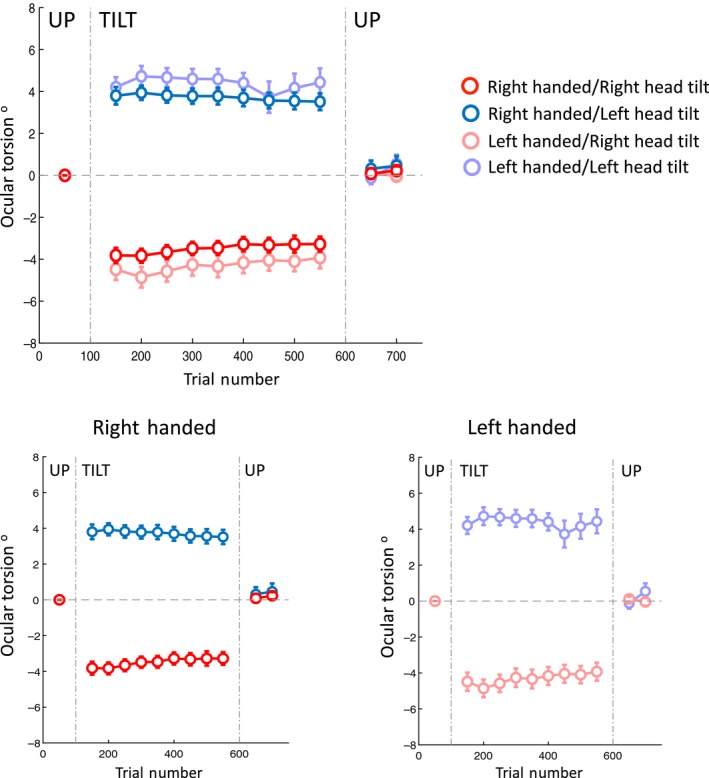
Average torsional eye position during head tilts to the right and left for both right‐handed and left‐handed subjects is shown together (top panel) and separately (bottom panels). As in the SVV plot, each point corresponds with the average ocular torsion within the blocks of 100 trials, and the gap in the data corresponds with the first 50 trials that were discarded. Error bars indicate SEM.

Table 1 and Figure 2 show the SVV results, and Table 2 and Figure 3 show the ocular torsion results during the experiment.

**Table 1:** Average SVV and precision values ± SEM (in degrees) for the right‐handed and left‐handed groups.

		SVV baseline	SVV tilt onset	SVV drift	SVV aftereffect	Precision baseline	Precision during tilt	Precision aftereffect
Right‐handed	Left tilt	−0.2 ± 0.3°	3.0 ± 1.3°	−5.2 ± 1.4^o^	−3.7 ± 0.5°	1.5 ± 0.2°	4.5 ± 0.4°	0.7 ± 0.2°
	Right tilt		−4.7 ± 1.5°	3.8 ± 1.5°	3.0 ± 0.7°			
Left‐handed	Left tilt	0.0 ± 0.4°	3.4 ± 1.1°	−3.8 ± 1.2°	−2.8 ± 0.5°	1.9 ± 0.3°	4.0 ± 0.4°	0.6 ± 0.3°
	Right tilt		−4.1 ± 1.0°	4.9 ± 1.7°	2.2 ± 0.5°			

**Table 2:** Average ocular torsion values ± SEM (in degrees) for the right‐ and left‐handed groups.

		Torsion tilt onset	Torsion drift	Torsion aftereffect
Right‐handed	Left tilt	3.8 ± 0.4°	−0.5 ± 0.4°	0.4 ± 0.4°
	Right tilt	−3.8 ± 0.4°	0.8 ± 0.3°	0.07 ± 0.25°
Left‐handed	Left tilt	4.2 ± 0.5°	−0.5 ± 0.6°	0.2 ± 0.4°
	Right tilt	−4.5 ± 0.5°	1.2 ± 0.4°	0.04 ± 0.16°

### SVV in the upright position

At the baseline upright position, an initial SVV value was calculated from the first 100 trials. In the right‐handed group (*n* = 22), there was an average SVV error of −0.2 ± 0.3° (mean ± SEM) before the head was tilted (Table 1 and Fig. 2). Separating the left and right head tilt sessions, the average SVV responses during the baseline upright position were −0.4 ± 0.5° and 0.1 ± 0.4°, respectively, and not significantly different (*t*‐test *P* = 0.3). In the left‐handed group (*n* = 22), there was an average SVV of 0.0 ± 0.4° at the baseline upright positions before the head was tilted (Table 1 and Fig. 2). Separating the left and right head tilt sessions, the average SVV responses during the baseline upright position were −0.3 ± 0.5° and 0.3 ± 0.5°, respectively, and not significantly different (*t*‐test *P* = 0.3). There was no significant difference in the baseline SVV values between right‐handed and left‐handed groups (*t*‐test *P* = 0.7).

### SVV at the beginning of the tilt

At the beginning of the head tilt, an initial bias in SVV responses was calculated from the first 100 trials (Table 1 and Fig. 2). In the right‐handed group, the average SVV at the beginning of the left head tilt was 3.0 ± 1.3°, and the average SVV at the beginning of the right head tilt was −4.7 ± 1.5°. The asymmetry, however, was not significant (i.e., there was no difference in absolute values; paired *t*‐test *P* = 0.3). During this initial tilt period, the majority of the right‐handed subjects had an SVV bias away from the head tilt (i.e., the E‐effect): 77.3% (*n* = 17) with the left tilt and 68.2% (*n* = 15) with the right tilt. In the left‐handed group, the average SVV at the beginning of the left head tilt was 3.4 ± 1.1° and the average SVV at the beginning of the right head tilt was −4.1 ± 1.0°. This asymmetry was nonsignificant (paired *t*‐test *P* = 0.6). During this initial tilt period, similar to the right‐handed group, the majority of the left‐handed subjects had an E‐effect: 72.7% (*n* = 16) with the left tilt and 90.9% (*n* = 20) with the right tilt. Handedness had neither a significant effect on the SVV values at the beginning of the tilt (repeated measures ANOVA, *P* = 0.6), nor had a significant effect on how head tilt changed these SVV values (interaction between the handedness and head tilt; repeated measures ANOVA, *P* = 0.9).

### Ocular torsion at the beginning of the tilt

Usable torsion measurements were obtained from 39 subjects (Table 2 and Fig. 3). The ocular torsion for all subjects was in the opposite direction of the head tilt (i.e., the OCR). For the right‐handed group, the average initial ocular torsion with the left and right head tilts were 3.8 ± 0.4° and −3.8 ± 0.4°, corresponding with ocular torsion gains of 0.2 in both directions (ocular torsion/head tilt). For the left‐handed group, the average initial ocular torsion with the left and right head tilts were 4.2 ± 0.5° and −4.5 ± 0.5°, corresponding with ocular torsion gains of 0.2 in both directions. There was no significant asymmetry in the initial ocular torsion between the right and left head tilts for either the right‐handed (paired *t*‐test *P* = 0.4, *n* = 19) or the left‐handed (paired *t*‐test *P* = 0.5, *n* = 20) group. Handedness had neither a significant effect on the ocular torsion at the beginning of the tilt (repeated measures ANOVA, *P* = 0.5), nor had a significant effect on how head tilt changed these ocular torsion values (interaction between the handedness and head tilt; repeated measures ANOVA, *P* = 0.33).

### SVV drift

SVV responses did not remain stable during the head tilt. That is, with a head tilt to the right, SVV drifted toward the right side, and with the head tilt to the left, it drifted toward the left side (Table 1 and Fig. 2). Overall, the SVV drift was toward the direction of the head tilt for both groups: In the right‐handed group, 86.4% (*n* = 19) with the left tilt, and 81.8% (*n* = 18) with the right tilt; in the left‐handed group, 77.3% (*n* = 17) with the left tilt, and 81.8% (*n* = 18) with the right tilt. By approximating the drift as a linear function, an average drift was determined as the slope of the linear fit to the data from all subjects. In the right‐handed group, the average SVV drift during 500 trials (~15 min) was −5.2 ± 1.4° for the left head tilt (*P* = 0.002) and 3.8 ± 1.5° for the right head tilt (*P* = 0.02). In the left‐handed group, the average SVV drift was −3.8 ± 1.2° for the left head tilt (*P* = 0.005) and 4.9 ± 1.7° for the right head tilt (*P* = 0.008). In both groups, the drift was symmetrical; i.e., there was no difference between the absolute drift values for the right and the left tilts (paired *t*‐test, right‐handed *P* = 0.5; left‐handed *P* = 0.4). Handedness had neither a significant effect on the SVV drifts (repeated measures ANOVA, *P* = 0.6), nor had a significant effect on how the head tilt changed the SVV drifts (interaction between the handedness and head tilt; repeated measures ANOVA, *P* = 0.9).

### Ocular torsion drift

The ocular torsion also drifted toward the direction of the head tilt in the majority of subjects (Table 2 and Fig. 3): In the right‐handed group, 79% (*n* = 15) with the left tilt, and 62% (*n* = 13) with the right tilt; in the left‐handed group, 62% (*n* = 13) with the left tilt, and 75% (*n* = 15) with the right tilt. In the right‐handed group, the average ocular torsion drift during 500 trials (~15 min) was −0.5 ± 0.4° for the left head tilt and 0.8 ± 0.3° for the right head tilt. In the left‐handed group, the average drift for the ocular torsion was −0.5 ± 0.6° for the left head tilt and 1.2 ± 0.4° for the right head tilt. In both the right‐handed and left‐handed groups, only the drifts of ocular torsion with the right tilt were significantly different from zero (*t*‐test right‐handed, left head tilt *P* = 0.3; right‐handed, right head tilt *P* = 0.009; left‐handed, left head tilt *P* = 0.4; left‐handed, right head tilt *P* = 0.01), but they were symmetrical with respect to the left tilt (i.e., no significant asymmetry, paired *t*‐test: right‐handed *P* = 0.7; left‐handed *P* = 0.3). Handedness had neither a significant effect on the ocular torsion drifts (repeated measures ANOVA, *P* = 0.6), nor had a significant effect on how head tilt changed the torsion drifts (interaction between the handedness and head tilt; repeated measures ANOVA, *P* = 0.7).

### SVV aftereffect

Once the head returned to the upright position, there was an aftereffect with respect to the baseline pre‐tilt SVV errors in the upright position (Table 1 and Fig. 2). There was a significant difference in the post‐tilt SVV compared with the baseline pre‐tilt SVV errors (i.e., SVV aftereffect, paired *t*‐test for all the four groups *P* < 0.001). In the right‐handed group, the average SVV aftereffect was −3.7 ± 0.5° following the left head tilt, and 3.0 ± 0.7° following the right head tilt, with no significant asymmetry (paired *t*‐test, *P* = 0.4). In the left‐handed group, the average SVV aftereffect was −2.8 ± 0.5° following the left head tilt, and 2.2 ± 0.5° following the right head tilt, with no significant asymmetry (paired *t*‐test, *P* = 0.4). Handedness had neither a significant effect on the SVV aftereffects (repeated measures ANOVA, *P* = 0.9), nor had a significant effect on how the head tilt changed the SVV aftereffects (interaction between the handedness and head tilt; repeated measures ANOVA, *P* = 0.2).

### Ocular torsion aftereffect

There were also aftereffects in ocular torsion once the head returned to the upright position (Table 2 and Fig. 3). In the right‐handed group, the average aftereffect in ocular torsion was 0.4 ± 0.4° after the left head tilt and 0.07 ± 0.25° after the right head tilt. In the left‐handed group, the average aftereffect in ocular torsion was 0.2 ± 0.4° after the left head tilt and 0.04 ± 0.16° after the right head tilt. In both right‐handed and left‐handed groups, the ocular torsion aftereffects were not significantly different from zero (*t*‐test right‐handed, left head tilt *P* = 0.4; right‐handed, right head tilt *P* = 0.8; left‐handed, left head tilt *P* = 0.88; left‐handed, right head tilt *P* = 0.8) and there was no asymmetry between the right and left head tilts (paired *t*‐test, right‐handed *P* = 0.8; left‐handed *P* = 0.8). Handedness had neither a significant effect on the ocular torsion aftereffects (repeated measures ANOVA, *P* = 0.90), nor had a significant effect on how head tilt changed the torsion aftereffects (interaction between the handedness and head tilt; repeated measures ANOVA, *P* = 0.96).

### SVV precision

Overall, the average SVV precision at the baseline upright position was not different between the right‐handed and left‐handed groups (Table 1) (*t*‐test, *P* = 0.3). In both groups, the SVV precision was worse while the head was tilted (average precision of right and left head tilts, right‐handed 4.5 ± 0.4°; left‐handed 4.0 ± 0.4°), compared with the baseline in upright position (right‐handed 1.5 ± 0.2°; left‐ handed 1.9 ± 0.3°; paired *t*‐test, in both right‐handed and left‐handed *P* < 0.00001). There was no significant drift in the SVV precision during the head tilts (*t*‐test right‐handed *P* = 0.6; left‐handed *P* = 0.05). The precision was also worse when the head returned to the upright position (right‐handed 4.3 ± 0.5°; left‐handed 4.3 ± 0.4°) compared with the baseline in upright position (paired *t*‐test, both right‐handed and left‐handed *P* < 0.00001). Handedness had no significant effect on the precision of SVV during head tilt, or after the head returned to the upright position (repeated measures ANOVA, precision of SVV during head tilt, *P* = 0.5; precision of SVV post‐tilt; *P* = 0.8). In addition, handedness did not significantly affect how the precision of SVV changed during or after the head tilt (interaction between handedness and head tilt, repeated measures ANOVA; precision of SVV during head tilt, *P* = 0.5; precision of SVV post‐tilt: *P* = 0.5). In both the right‐handed and left‐handed groups, we tested whether there was a relationship between the SVV precision and the SVV drift or aftereffect, but there was no significant correlation between any of these measurements (*P* > 0.1 in all comparisons). These results suggest that the drift in SVV was not related to the lack of attention or fatigue.

### Reaction time

Overall, the average reaction time at the baseline upright position was not different between the right‐handed and left‐handed groups (right‐handed 0.65 ± 0.02 sec; left‐handed 0.67 ± 0.02 sec; *t‐*test, *P* = 0.4), and in both groups the mean reaction time was longer while the head was tilted compared with the baseline in upright position (right‐handed 0.72 ± 0.02 sec; left‐handed 0.70 ± 0.02; paired *t*‐test, right‐handed *P* < 0.00001, left‐handed *P* = 0.05). Handedness had no significant effect on the reaction times during the head tilts (repeated measures ANOVA, *P* = 0.6). We also tested whether there was a correlation between the reaction time and SVV precision. In both right‐handed and left‐handed groups, the correlation between reaction time and SVV precision was nonsignificant during the head tilts in either direction (*P* > 0.4 in all four comparisons).

## Discussion

The direction of gravity is a fundamental frame of reference for quantifying orientation in space. Accordingly, upright perception has been widely used to study the effects of head or body position on spatial orientation (Wade 1968; Young et al., 1975; de Graaf et al., 1992; Guerraz et al., 1998; Tarnutzer et al., 2010; Alberts et al., 2016; Kheradmand and Winnick 2017). When the head is tilted laterally, the low gain of ocular counter‐roll is a source of error in spatial orientation. Here, we measured ocular torsion and SVV responses simultaneously with changes in the lateral head tilt position. Both right‐handers and left‐handers showed the typical bias in perception of upright orientation during 20° lateral head tilts (i.e., the E‐effect). There were no significant differences between the two groups in the accuracy or precision of SVV responses, SVV drift during head tilt, or SVV aftereffect when the head was brought back to the upright position. These findings show that the effect of head tilt on spatial orientation is comparable between right‐handers and left‐handers. Similar to the SVV results, changes in ocular torsion did not differ between the right‐handed and left‐handed groups. Taken together, these results could be interpreted with respect to hemispheric asymmetries and spatial orientation:

### Lateral head tilts and SVV

Perception of upright requires integration of the visual inputs from the retina, graviceptive signals from the otoliths, and proprioceptive inputs that encode the eye, head, and body positions. In this context, the SVV error during head tilt reflects the function of neural processes involved in multisensory integration for spatial orientation. If there was a functional laterality in these neural processes analogous to motor control, a systematic bias in spatial orientation would be expected based on the direction in which the head is tilted in space. The fact that the SVV errors did not differ between right‐handers and left‐handers, or between the head tilt directions in each group, shows such functional laterality does not exist in perception of spatial orientation. From an ecological standpoint, this is not surprising, as with changes in the head position, a non‐biased spatial perception is crucial for effective motor planning and interaction with the surrounding environment. In this view, while hemispheric asymmetry heavily influences motor planning and execution, perception of spatial orientation should not be biased by the changes in the head and eye positions. Such lack of functional laterality, however, cannot be interpreted as definitive evidence against asymmetric hemispheric contribution to the perception of spatial orientation. Non‐biased spatial orientation could still be generated through unequal right and left hemispheric contributions, as shown consistently by lesion studies (De Renzi et al., 1971; Kerkhoff and Zoelch, 1998; Kerkhoff 1999; Suzuki et al., 2001; Gentaz et al., 2002; Saj et al., 2005; Karnath and Dieterich 2006; Funk et al., 2011; Utz et al., 2011; Karnath and Rorden 2012; Braem et al., 2014). Stroke studies, in particular, have found that the right hemisphere lesions tend to have greater impact on perception of upright, a finding which indicates a dominance of the right hemisphere for processing spatial information in right‐handers (Bonan et al., 2006; Pérennou et al. 2008; Baier et al., 2012; Piscicelli et al., 2015). Left‐handers, however, are routinely excluded or underrepresented in these studies, and thus their hemispheric contributions to spatial orientation remain uncertain.

Consistent with the findings from previous studies, here SVV responses did not remain stable and there was a drift toward the direction of the head tilt (Wade 1968; 1970; Tarnutzer et al., 2013; Otero‐Millan and Kheradmand 2016; Kheradmand and Winnick 2017). In addition, there was a corresponding aftereffect when the head returned to upright position. Both of these findings reflect adaptive changes in perception of spatial orientation during head tilt (Wade 1968; 1970; Wade and Day 1968; Tarnutzer et al., 2013; 2014; Otero‐Millan and Kheradmand 2016; Kheradmand and Winnick 2017; Otero‐Millan et al., 2018). The SVV drift and aftereffect did not differ between right‐handers and left‐handers, or between the two head tilt directions in each group. These results, again, show no effect of handedness‐related hemispheric asymmetry on the neural processes that modulate spatial orientation during and after lateral head tilts. There was also no difference in the precision of SVV responses between right‐handers and left‐handers, or between the two head tilt directions in each group. In addition, we found no correlation between the SVV precision and SVV drift or SVV aftereffect. These findings show that the drift and aftereffect were not affected by fatigue or possible attentional shifts during the SVV task.

### Lateral head tilts and ocular torsion

During static head tilt, vestibular inputs from the otolith organs maintain torsional eye position in the opposite direction of the head tilt, partially compensating for the change in the head position relative to gravity (Leigh and Zee 2015). Similar to the SVV results, here we found no difference in ocular torsion during head tilt between right‐handers and left‐handers. This finding shows no effect of handedness‐related brain asymmetries on the vestibulo‐ocular responses during lateral head tilts. In addition, while the average drift and aftereffect in ocular torsion were in the same direction of the head tilt, they were much smaller and less consistent across head tilts than the SVV drift and aftereffect. In line with previous studies, these results show that the torsional eye position – or its driving input from the otoliths – is not the source of adaptive changes in spatial orientation during head tilt (Otero‐Millan and Kheradmand 2016; Otero‐Millan et al., 2018).

In conclusion, here we studied functional laterality in spatial orientation by tracking upright perception and ocular torsion. We found that the measures of upright perception and ocular torsion did not differ between right‐handers and left‐handers during head tilts or in the upright position before and after head tilts. These findings show no functional laterality, neither in the higher level neural mechanisms that maintain spatial orientation, nor in the lower level mechanisms that change ocular torsion during lateral head tilt.
